# Assessment of Morphologic Change of Mandibular Condyle in Temporomandibular Joint Osteoarthritis Patients with Stabilization Splint Therapy: A Pilot Study

**DOI:** 10.3390/healthcare10101939

**Published:** 2022-10-03

**Authors:** Tae-Hoon Kim, Youn Joong Kim, Yun-Heon Song, Ilho Tae, Ho-Kyung Lim, Seok-Ki Jung

**Affiliations:** 1 Department of Orthodontics, Graduate School of Clinical Dentistry, Korea University, Seoul 02841, Korea; 2 TMJ & Orofacial Pain Center, Ahrim Dental Hospital, Seoul 06169, Korea; 3 Department of Oral and Maxillofacial Surgery, Korea University Guro Hospital, Seoul 08308, Korea; 4 Department of Orthodontics, Korea University Guro Hospital, Seoul 08308, Korea

**Keywords:** temporomandibular joint osteoarthritis, stabilization splint, cone-beam computed tomography, shape correspondence analysis

## Abstract

(1) Background: The purpose of this study was to evaluate the 3-dimensional bony changes of the mandibular condyle in temporomandibular joints-osteoarthritis (TMJ-OA) patients treated with stabilization splint (SS) therapy using shape correspondence analysis. (2) Methods: A total of 27 adult patients (2 men and 25 women) with a mean age of 24.6 ± 3.9 years were included in this study. All patients were diagnosed with TMJ-OA and were treated with an SS. Cone-beam computed tomography data of the condylar head before and after SS therapy from 42 condyles (15 bilateral and 12 unilateral TMJ-OA) were used for the analysis. For the performance shape correspondence analysis (SPHARM-PDM), statistical differences were performed using the one-way analysis of variance and Scheffe post hoc tests. (3) Results: After SS treatment in TMJ-OA patients, bone resorption of the condyle head surface was predominant in the anterosuperior, superolateral, and superior areas, and bone formation was superior in the lateral, medial, posterosuperior, and posteromedial areas. The change in the condylar volume between the two groups was not statistically significant. (4) Conclusions: After SS treatment in TMJ-OA patients, there was both bone resorption and bone formation on the mandibular condyle head surface, which induced morphological changes in the condyle head.

## 1. Introduction

Temporomandibular disorder (TMD) is a collective term for clinical disorders involving the temporomandibular joint (TMJ), masticatory muscles, and related structures [1]. TMD tends to be more common in women and affects about 10% of the total population [2]. TMD may be related to many causes, such as injury, dysfunctions, or neoformations [3]. Persistent pain and mouth opening restrictions associated with TMD can lead to severe chronic pain and dysfunction [4]. Prolonged TMD progresses to TMJ osteoarthritis (OA), a severe form of TMD characterized by degenerative bone changes accompanied by inflammation [5]. A major cause of TMJ-OA is excessive overload of the joint that exceeds physiological tolerance. This joint overload leads to degenerative changes in the TMJ surface and consequent cortical bony destruction [6]. Sometimes, the resulting overall morphological changes, such as deviations, disc displacements, adhesions, and osteoarthritic processes can occur with or without pain or dysfunction [7].

Physical therapy, drug therapy, occlusal device therapy, and intra-articular injection are common conservative treatment methods for TMJ-OA [8,9,10]. Among these treatment options, the stabilization splint (SS) has been clinically reported to effectively protect the TMJ from occlusal overload, relieve excessive muscle tension, and reduce the destructive power of bruxism [6]. In fact, there are reports that the use of SS improved the clinical symptoms of severe TMJ arthralgia [11,12]. However, although there is a great clinical need for more studies on the effects of SS in OA patients, the effect has not been fully elucidated. In addition to the clinical improvement due to SS, radiographic changes through SS use should also be identified, considering the degenerative bone changes of the condyle due to TMJ-OA [6].

Liu et al. [13] reported a bone formation in the medial and intermediate segments of the condyle when an anterior repositioning splint (ARS) was applied using computed tomography (CT) images. Ok et al. [14] found that their SS group exhibited a higher ratio of bone formation in the anterior division of the condyle. They also found higher frequencies of cortical thickening in the anterior and posterior sections of the condyle compared to that seen in the non-SS group, identified using cone-beam computed tomography (CBCT) images.

CBCT is generally accepted as the most useful technique for assessing OA changes in the TMJ as it provides clinicians and researchers with detailed information on bone remodeling. It produces a clear visualization of the hard tissues of the TMJ and markedly reduces radiation and cost compared to multi-slice spiral CT [15]. However, previous studies did not report changes in the corresponding anatomic regions. The standardization of cross-sections in the assessment of multiplanar images is challenging in longitudinal and cross-participant studies.

Recently, Paniagua et al. introduced a novel method using CBCT and 3-dimensional (3D) structural and statistical spherical harmonic statistical shape analysis (SPHARM-PDM) to quantify TMJ-OA [16]. This innovative method for quantifying TMJ morphologic changes minimizes the importance of the examiner’s experience, reduces intra-and inter-rater-related errors, standardizes findings, allows new discoveries, and contributes to the development of new imaging markers for risk factors [17].

The purpose of this study was to evaluate the 3D bony changes of the mandibular condyle head in TMJ-OA patients treated with SS therapy using shape correspondence analysis.

## 2. Materials and Methods

### 2.1. Participants

This retrospective study used 3D surface mesh models of mandibular condyles from CBCT images of patients diagnosed with TMJ-OA. The participants were selected from adult patients aged over 20 years, who visited the local clinic for TMD treatment from April 2008 to September 2016, and who underwent 3D CBCT (DCT-90-P, Vatech, Korea: 50–90 KvP, 2–10 mA, 24 s, 0.2 mm voxel size, field of view, 120 × 85 mm) as routine diagnostic records. Patients were seated and asked to rest their heads at the center of the proprietary headrest. The patients were instructed to bite their teeth in the intercuspal position. The center beam was aligned with the sagittal plane, and the seat position was adjusted so that the lateral crossed cursor was targeted at the condyle. The scanned data were automatically exported to the DICOM format.

SS therapy was provided to all patients, and the patients who had restricted mouth opening with TMJ closed lock had it reduced by manual manipulation before wearing the splint. The splints were fabricated using acrylic resin on the lower arch and with the mandible guided to a centric position.

CBCT images were taken before (T1) and after SS therapy (T2) when signs of TMJ-OA were not detected on at least two series of CBCT images. Patients who were diagnosed with TMJ-OA on only one side were classified as unilateral, and those with both sides affected were classified as bilateral.

The exclusion criteria were (1) systemic medical conditions involving the immune system, degenerative musculoskeletal system, or neuropathy sequelae; (2) craniofacial syndromes; (3) facial trauma related to the TMJ; and (4) history of orthodontic treatment, orthognathic surgery, or TMD treatment, including splint therapy. This study was approved by the Institutional Review Board of the Korea University Guro Hospital (No. 2022GR0231).

### 2.2. Image Preparation

All scan images were reformatted to a voxel size of 0.5 mm^3^ to standardize the voxel size and decrease the computational power and time required to compute the automated registration using open-source software (3D Slicer v.4.8.0, http://www.slicer.org (accessed on 1 June 2021)), and 3D surface mesh models of the right and left mandibular condyles were constructed using semiautomatic discrimination procedures that outlined the cortical boundaries of the condylar region and allowed manual editing, checking slice-by-slice in all three planes of space using open-source software (ITK-SNAP software v.3.6.0, http://www.itksnap.org (accessed on 1 June 2021)) [18]. After generating all 3D surface models, the left condyles were mirrored in the sagittal plane to form the right condyles to facilitate comparison.

**Figure 1:**
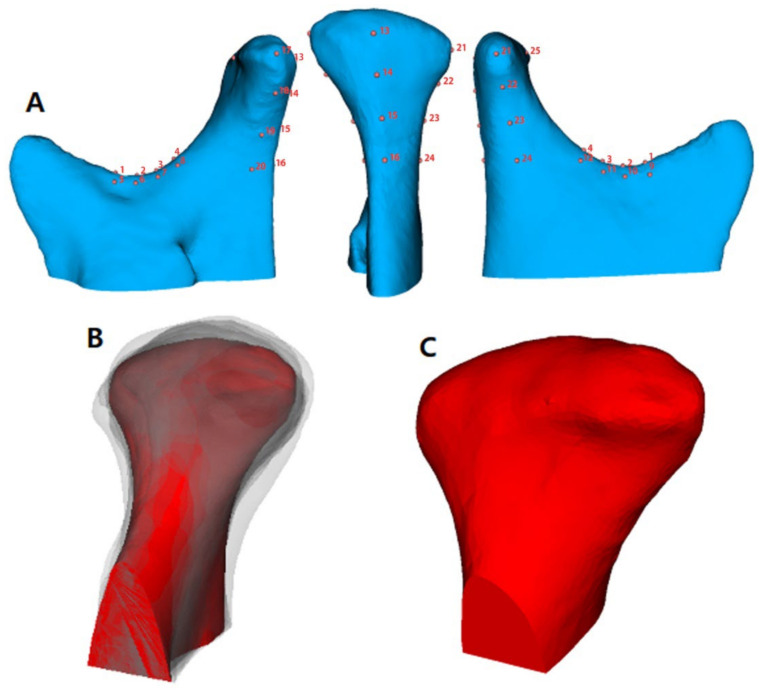
(**A**), 25 landmark registration for surface model approximation; (**B**), transparent overlays of the condyles approximated at the same coordinate; (**C**), cropped final surface model.

**Figure 2:**
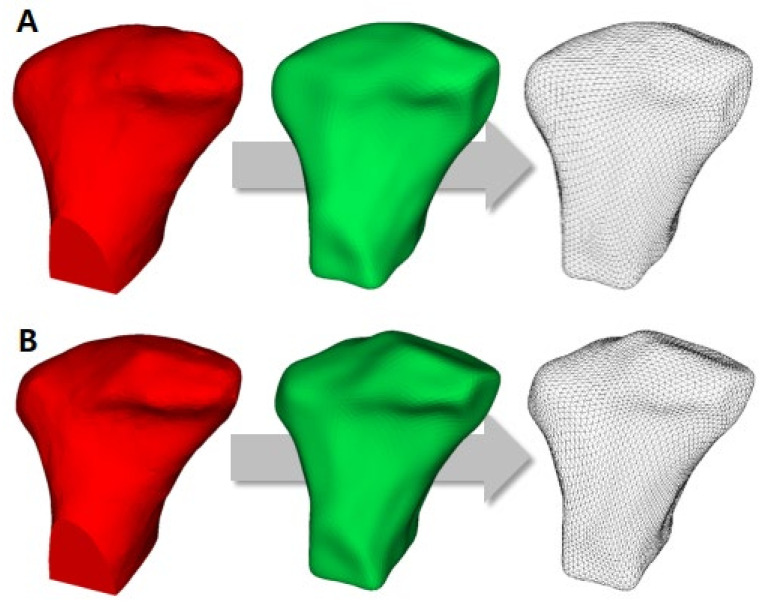
The left condyle model represents the surface model for the condyle cropped by region of interest. The middle condyle model represents the SPHARM-PDM model from the original mesh surface model. The right condyle model represents the final surface mesh model for the shape correspondence analysis. (**A**), pre-treatment (T1) model; (**B**), post-treatment (T2) model.

Because of the individual morphological variability across participants, a landmark-based approach was used to consistently approximate all the condyles in the same coordinate system. The 25 landmarks used in a previous study [19] were placed on each condylar surface model, and landmarks were used only for the spatial approximation of all the condyles in a common arbitrary XYZ coordinate system (Figure 1). In other words, the 25 landmarks were used only for the registration of all surface models and not for the analysis of morphological variability. After registration, all 3D surface models were simultaneously clipped/cropped to define the condylar region of interest (ROI), and shape correspondence analysis (SPHARM-PDM, 3D Slicer v.4.8.0) was used to generate a mesh approximation from the volumes whose points were mapped to a spherical map (Figure 2). The homology/correspondence of the mapping of the 4002 points across all participants was verified using color-coded maps of surface parameterization.

Shape correspondence made it possible to mark the ROI in one condyle and propagate such regions for the other time points, obtaining the X, Y, and Z coordinates for each point (Pick ’n Paint, 3D Slicer v.4.8.0). Then, mathematical formulas were applied to each coordinate of the corresponding points on the condylar surface, which allowed the measurement of the condylar surface changes pre- and post-treatment.

**Figure 3:**
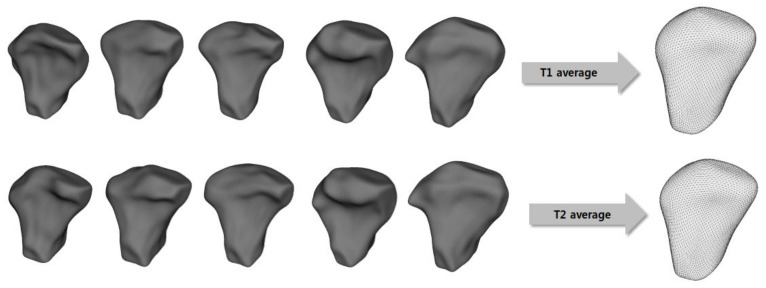
The creation process for the group average surface mesh model for pre-treatment (T1) and post-treatment (T2) analysis.

After creating each condyle surface model for shape correspondence analysis, a group average mesh model was created for visualization of the comparison between the T1 and T2 groups (Shape Variation Analyzer, 3D Slicer v.4.8.0) (Figure 3).

### 2.3. Measurements

**Figure 4:**
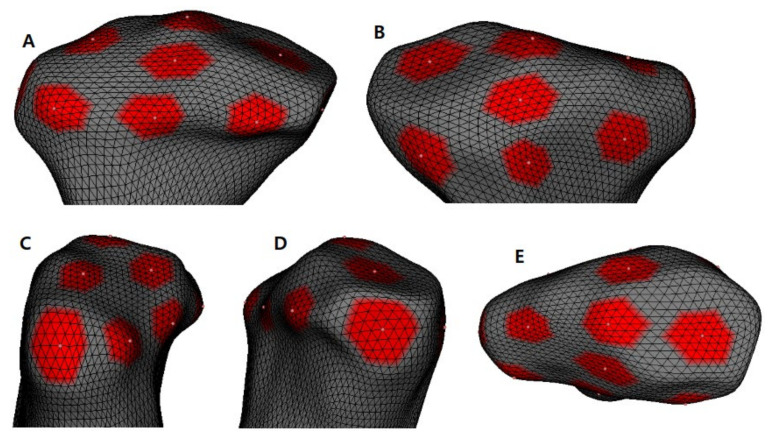
Close-up view of the areas used for the mesh statistics; (**A**), anterior view; (**B**), posterior view; (**C**), lateral view; (**D**), medial view; (**E**), superior view.

**Figure 5:**
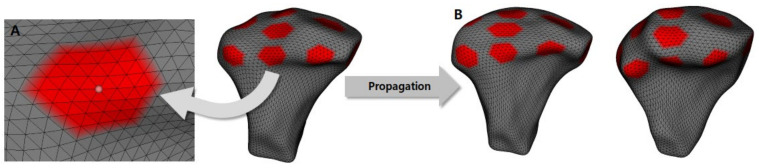
(**A**), 37 points per area for the mesh statistics; (**B**), examples of mesh propagation. All condyle models utilized the same area for the mesh statistics.

The following 13 specific ROIs were selected as representatives of each condylar surface (Figure 4): anterior (A), superior (S), posterior (P), lateral (L), medial (M), anteromedial (AM), anterolateral (AL), posterolateral (PL), posteroanterior (PA), superomedial (SM), superolateral (SL), anterosuperior (AS), and posterosuperior (PS). Thirty-seven corresponding points were used to analyze the changes in each ROI (Figure 5). The referred ROIs were marked in the pre-treatment 3D models and propagated to the respective post-treatment models (Pick ’n Paint, 3D Slicer v.4.8.0), and 3D pointwise linear distances between each time point were then used to calculate remodeling changes in millimeters (model-to-model distance, 3D Slicer v.4.8.0). The condylar volume changes between T1 and T2 were also calculated in cubic millimeters using ITK-SNAP software.

### 2.4. Statistical Analysis

The differences among the ROIs for the condylar surface changes were analyzed using one-way analysis of variance with a post hoc test (Scheffe). To compare volume change between T1 and T2, statistical analysis based on an independent *t*-test was used. A significance level of *p* ≤ 0.05 was applied. Statistical analyses were performed using SPSS version 24.0 software (SPSS Inc., Chicago, IL, USA).

## 3. Results

A total of 27 adult patients (2 men and 25 women) with a mean age of 24.6 ± 3.9 years were included in this study. Fifteen patients had bilateral TMJ, and 12 patients had unilateral TMJ-OA involvement. Finally, data from 42 condyles were used in the analysis of the condylar surface and volume changes. The average duration of SS treatment was 2.5 ± 1.3 years (Table 1).

**Figure 6:**
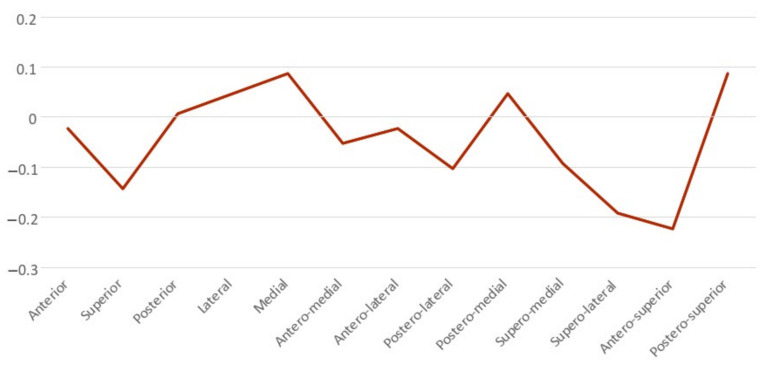
Means plots of the condylar surface changes on each surface area.

After SS treatment, bone resorption of the mandibular condylar head occurred at the AS, SL, S, PL, SM, AM, A, and AL areas, and bone formation of the mandibular condylar head was observed at the P, PM, L, M, and PS areas of the mandibular condyle head. The predominant areas for bone resorption were the AS and SL and those for bone formation were the PM, L, M, and PS (Figure 6, Table 2 and Table 3).

**Figure 7:**
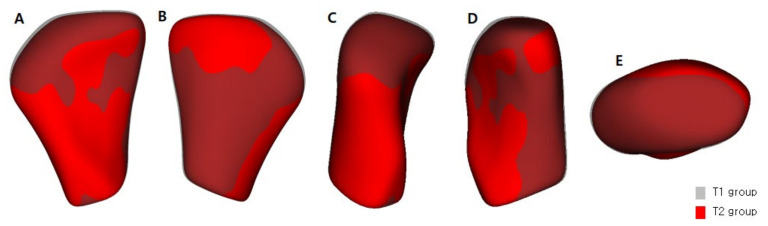
Group comparison using shape analysis—semi-transparent overlays pre-treatment (T1) and post-treatment (T2); (**A**), anterior view; (**B**), posterior view; (**C**), lateral view; (**D**), medial view; (**E**), superior view.

**Figure 8:**
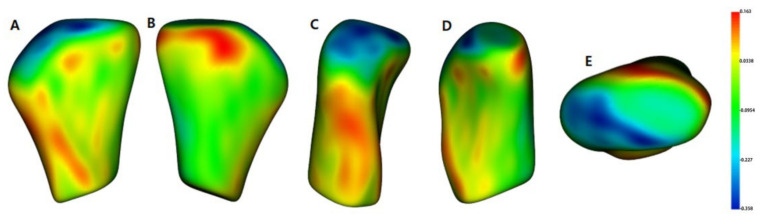
Group comparison using shape analysis—color maps pre-treatment (T1) and post-treatment (T2); (**A**), anterior view; (**B**), posterior view; (**C**), lateral view; (**D**), medial view; (**E**), superior view.

**Figure 9:**
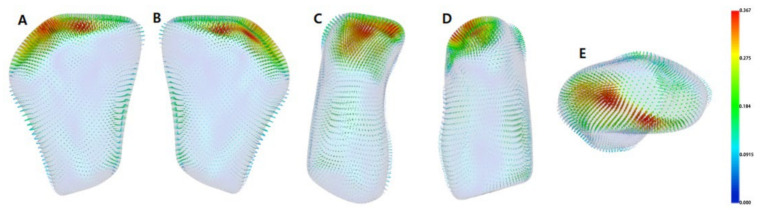
Group comparison using shape analysis—vector maps pre-treatment (T1) and post-treatment (T2); (**A**), anterior view; (**B**), posterior view; (**C**), lateral view; (**D**), medial view; (**E**), superior view.

The semi-transparent overlays visualized the bone resorption and formation of the condyle head by comparing the groups’ average surface model (Figure 7). The signed distance difference was determined using color maps to show the magnitude of the surface changes (Figure 8). In the color maps, blue areas indicate bone resorption, and red areas indicate bone formation of the mandibular condyle head. Vector maps (Figure 9) show the direction of bony changes on the mandibular condyle head surface. The condylar volume changes after SS treatment were not statistically significant (Table 4).

## 4. Discussion

In the present study, TMJ-OA patients showed bone surface remodeling after SS therapy, especially in the AS, SL, and S areas for bone resorption and in the L, M, PS, and SM areas for bone formation. A previous study showed similar results for the mandibular condyle bone surface remodeling. Liu et al. [13] reported bone formation of the mandibular condyle head after ARS therapy in the P bevel and M and middle parts of the condyles. Ok et al. [14] also reported bone formation and cortical thickening in TMJ-OA patients after SS treatment using CBCT. Yano et al. [19] reported that ARS therapy not only repositioned the displaced articular disc but also induced condylar bone remodeling and presented a double contoured condylar head on magnetic resonance imaging (MRI) as evidence.

A double-contour condylar head was first reported by Hollender et al. in 1974 [20,21]. They observed clear double contours posterior and superolateral of the condylar head after osteotomy for the treatment of mandibular ramus and condylar fractures. These double contour images were also observed after other mandibular bone surgery [20], condylar trauma [21], splint treatment, Herbst appliance treatment [22], occlusal adjustment [23], and other conditions [24], which is due to adaptive bone remodeling of the condylar head. The double contour image is thought to be the result of bone remodeling that appears in the adaptation process due to stress changes in the joint space [20,21,22,24]. In this study, a double contour was also observed in the posterior of the condyle, which was similar to the previous study [13,14].

Suei et al. [24] histologically verified the presence of double contour formations in the human mandibular condylar head. Since no cartilage layer was found on the condylar surface histologically, they suggested that the double contour formation was due to periosteal reaction. However, other studies have suggested that the double contours of the condyle and posterior ramus are the result of endochondral and periosteal bone formation [22]. Similar condylar remodeling was also observed in rats that attempted treatment of mandibular protrusions using a fixed occlusal jumping device [23]. In that study, bone deposition occurred on the P and S surfaces of the rat condyle but was rarely observed on the A surface. They reported that the mandibular anterior repositioning improved the proliferation of mesenchymal cells in the rat condylar cartilage, especially the P surface.

Although disc changes can promote bone remodeling, stress changes in joint space due to splint-generated condylar repositioning are thought to be the major contributing factors. In a previous study, it was hypothesized that the release of pressure in the articular fossa would induce bone remodeling in the posterior part of the condyle [13,24]. Liu et al. [13] reported that downward and anterior movements of the condyle resulting from the use of ARS increase both posterior and medial articular space. Mandibular anterior and downward positioning with ARS prevents condyles from vascularizing and articulating with well-distributed retrodiscal tissue at the posterior attachment. Over time, the retrodiscal tissue undergoes a process of adaptation, and when the patient removes the ARS, the condyle moves posteriorly into the fossa to function in the adapted retrodiscal tissue, and the disc is still likely to be displaced to varying degrees [25]. The condylar function of the adapted posterior disc tissue may also promote bone remodeling by reducing joint load and stress. Similarly, Yoko et al. [26] found that, with or without TMJ pain, after application of SS, the condyles moved significantly forward and backward with rotation in the direction of the opening of the mouth. In addition, Ekberg et al. [27] and Demling et al. [28] also reported that the condyles were displaced anteriorly and inferiorly after SS application. Therefore, the bone formation tendency on the L, M, PS, and PM surfaces of the condyle head surface after SS therapy in the present study could be explained by the anterior and inferior movement of the condyle due to splint wearing.

In patients with TMJ-OA, functional and mechanical loading can occur on the A surface of the glenoid fossa and on the condyle head. These loads can affect the shape and change the surface of the glenoid fossa [29]. Elguy et al. [30] reported that there was a significant relationship between sagittal changes in the mandibular condyle and roof thickness of glenoid fossa in TMJ-OA patients. Ok et al. [31] evaluated the glenoid fossa for three categories: cortical bone integrity, sclerosis, and subchondral cysts after SS therapy in the non-SS group. They found that the non-SS group showed a significant decrease in distance from the inflection point, whereas the SS group showed an increase. This means that SS therapy can relieve excessive load on the mandible, cause adaptive remodeling, and maintain the lowest point distance from the articular eminence. Since only the mandibular condyle shape was considered in this study, further study is needed for three-dimensional evaluation of joint and morphological changes related to TMJ-OA and SS therapy, which were not evaluated in this study.

Morphological changes in the TMJ can be evaluated using various methods (panoramic and transcranial radiographs, conventional CT, CBCT, and MRI) [32]. However, panoramic and transcranial radiography have some limitations, such as structural distortion, overlap of the zygomatic process, and inability to display the entire articular surface of the TMJ [33]. Panoramic radiographs also have low sensitivity for detecting bony changes in TMJ [15]. Therefore, it has limited value in TMJ assessments. CT tomography can be used to evaluate the bone contours of the TMJ, but it is expensive and requires high radiation exposure. In contrast, CBCT is less expensive and has a lower radiation dose. In addition to these advantages, CBCT can provide a three-dimensional change to the TMJ. Indeed, CBCT has been shown to be effective in diagnosing several bone changes in TMJ [34].

In the present study, shape correspondence analysis (SPHARM-PDM) was employed to quantify morphological changes in the 3D condylar head during SS therapy. SPHARM-PDM is capable of providing a unique point-to-point correspondence on all measured surfaces [16]. This methodology uses the nearest point-to-surface distance to quantify change. Nearest point-to-surface measurement has been introduced in 3D image analysis because it can reduce operator bias during data collection and intra-inspector reliability errors. This method is fairly accurate when the ROI exhibits a small growth displacement. For participants with increased vertical or longitudinal growth in studies with long observation periods, repeated closest point measurements may misreport changes or underestimate the extent of displacement because the software algorithm often measures the nearest adjacent surface, rather than that surface. Although 3D point-to-point measurements can reduce these potential errors, they can still be affected by operator bias. Moreover, if the ROI is actively remodeling, such as in the condyle, selecting a certain point is not always reliable or reproducible. As opposed to finding specific points within an area, SPHARM-PDM can overcome many of these errors and biases by evaluating the entire surface and counting thousands of corresponding points [35]. Through this shape-matching technique, it was possible to quantitatively evaluate the morphological change of the mandibular condyle head surface after SS therapy in this study.

The present study has several limitations. First, the number of patients enrolled in the study was too small to be considered statistically significant. Further investigation with a sufficient number of samples is necessary to solve this problem. Secondly, there was no control group for comparison with the treatment group. This study was a retrospective study conducted on patients who visited the hospital for treatment for TMJOA. It was practically difficult to obtain T1 data from patients who came to the hospital for TMJOA treatment but returned without treatment. A prospective study including a control group needs to be conducted in the future. Symptomatic improvement in the patients, including pain, sound, maximum opening, and other markers, were not considered, although they are a significant factor in the evaluation of the treatment effects of most TMJ disorders. In addition, disk displacement types were not classified, although several studies have reported that the treatment effects were not statistically different among disk displacement types. In addition, we could not completely control for problems that could cause various errors such as imaging errors, program precision, and patient movement; regardless, this could be the factor that affected the treatment results. MRI evaluation is necessary to clarify the disk displacement types of patients. Therefore, further studies should include MRI investigations of morphological changes in condyles.

## 5. Conclusions

1. After SS treatment in TMJ-OA patients, there was both bone resorption and bone formation on the mandibular condyle head surface, which induced morphological changes in the condyle head.
2. Bone resorption of the mandibular condyle head surface was predominant in the AS, SL, and S areas, while bone formation was superior in the L, M, PS, and PM areas.
3. The mandibular condyle volume changes before treatment (T1) and after SS treatment (T2) were not statistically significant.

## Figures and Tables

**Table 1:** Patients’ characteristics (*n* = 27).

Variables	Values
Gender	
Female	25
Male	2
TMJ OA	
Bilateral	15
Unilateral	12
Age (years)	
Mean (range)	24.6 ± 3.9 (20.0–31.1)
Treatment duration (years)	
Mean (range)	2.5 ± 1.3 (1.3–7.9)

**Table 2:** Descriptive statistics for condylar surface changes between T1 and T2 group.

Condylar Morphologic Changes (mm)													
	A	S	P	L	M	AM	AL	PL	PM	SM	SL	AS	PS
Mean	−0.02	−0.14	0.01	0.05	0.09	−0.05	−0.02	−0.1	0.05	−0.09	−0.19	−0.22	0.09
SD	0.32	0.4	0.24	0.25	0.27	0.3	0.32	0.23	0.24	0.41	0.44	0.44	0.5
Min	−1.4	−1.57	−0.77	−0.90	−0.62	−1.5	−1.71	−1.04	−0.61	−1.14	−1.84	−1.84	−2.2
Max	0.62	0.55	0.65	1.02	1.08	0.53	0.65	0.32	0.92	0.61	0.72	0.72	0.54
Percentile													
25th	−0.12	−0.33	−0.12	−0.08	−0.08	−0.19	−0.13	−0.17	−0.09	−0.11	−0.36	−0.28	−0.06
75th	0.19	0.07	0.13	0.18	0.25	0.14	0.16	0.03	0.17	0.05	0.08	0.07	0.22

**Table 3:** Scheffe post hoc comparisons of condylar surface changes between T1 and T2 group *.

Group	1	2	3	4	5	6	7	8
AS	−0.22							
SL	−0.19	−0.19						
S		−0.14	−0.14					
PL			−0.10	−0.10				
SM				−0.09				
AM				−0.05	−0.05			
A					−0.02	−0.02		
AL					−0.02	−0.02		
P						0.01	0.01	
PM							0.05	0.05
L							0.05	0.05
M								0.09
PS								0.09
Sig.	0.882	0.264	0.561	0.208	0.905	0.898	0.428	0.305

* The number indicates the statistical relationship between sites. The same numbers indicate non-significant difference between sites (*p* > 0.05).

**Table 4:** Volume changes between T1 and T2.

Variable	T1 (Average ± SD)	T2 (Average ± SD)	*p* Value
Condylar volume, mm^3^	1182.64 ± 329.73	1149.54 ± 320.53	0.642

## Data Availability

The datasets generated and/or analysed during the current study are not publicly available but are available from the corresponding author on reasonable request.
